# Metabolites and Plant Hormones Related to the Resistance Response to Feeding Stimulation and Leaf Clipping Control in Chinese Pine (*Pinus tabuliformis* Carr.)

**DOI:** 10.3390/cimb45020072

**Published:** 2023-01-30

**Authors:** Yanan Zhao, Guona Zhou, Tianhua Sun, Lifeng Wang, Qiang Xu, Junxia Liu, Baojia Gao

**Affiliations:** Forestry College, Hebei Agricultural University, Baoding 071000, China

**Keywords:** feeding stimulation, flavonoid pathway, metabolome, plant hormone, resistant

## Abstract

This experiment was conducted to define changes in metabolic pathways in response to mandibulate insect feeding and to provide a reference for further investigation of the molecular mechanisms underlying the development of conifer resistance. Chinese pine (*Pinus tabuliformis* Carr.) in good growth status in natural condition was chosen for stimulation by 10 pine caterpillars (*Dendrolimus tabulaefomis* Tsai et Liu) as feeding stimulation (FS), leaf clipping control (LCC) as mechanical damage, and CK group (with no treatment) (recorded as 0 h). The metabolome and total flavonoid content were measured in the needles at 0, 2, and 8 h after treatment. Plant hormones were measured with needles at 0, 0.5, 1, 1.5, 2, 4, 6, and 8 h after different treatments. The results show that a total of 30.8% flavonoids are identified by metabolomics analysis. Compared with leaf clipping control, feeding stimulation of Chinese pine caterpillars significantly induced the upregulation of metabolites in the flavonoid pathway in Chinese pine, and the plant hormones JA and IAA showed expression trends consistent with those of the metabolome. According to the biological processes of the four plant hormones involved, JA and SA are mostly involved in resistance formation, and in this study, both of them also have fluctuating expressions influenced by feeding stimulation, while the expressions of the growth-related hormones IAA and ABA have no significant changes at other time points except for 1 h after treatment. Thus, the flavonoid pathway is one of the main pathways involved in resistance formation in conifers, and JA and IAA are involved in the formation of resistance.

## 1. Introduction

Plants exposed to stress activate the related jasmonic acid (JA), salicylic acid (SA), and ethylene (ET) pathways to enable resistance [1,2,3,4,5], and interactions among JA, methyl jasmonate (MeJA), SA, and abscisic acid (ABA) are widespread in existence [6,7]. Though plants initiate earlier resistance to pathogens through the SA pathway, the accumulation differences of substances in the SA pathway occur after many plants are infested with pathogens [8,9]; an insect also leads to the cooperative expression of the SA signaling pathway with the JA signaling pathway [10,11]. Generally speaking, chewing insects would induce JA/ABA coregulation, and sucking insects lead to JA, SA, and ET for response [12,13]. Jingjun Ruan [14] revealed that JA regulates the mechanism under insect stress. ABA is involved in the regulation of plant resistance by affecting the opacity and closure of stomata to reduce water loss, which in turn regulates photosynthesis [15,16]. Interrelationships in resistance conferred by the closely related hormones JA and SA and growth- and development-related hormones during resistance formation remain unclear in tree-insect interaction. Many changing pathways—such as the flavonoid [17,18,19,20], citric acid metabolism [21], and glutathione metabolism [22,23,24] pathways—in the stress response process have been discovered in a joint analysis of the transcriptome and metabolome, in which the most accumulated substances have been further studied to identify key genes regulating traits and resistance to be applied in breeding [25,26]. Different stressors induce changes in metabolic pathways, companying plant hormone signaling. In plants, SA can be synthesized through phenylalanine and isobranchic acid, both of which have mangiferic acid as a precursor [27,28]. IAA can be generated from L-tryptophan and indole in the tryptophan pathway. Most of the carbon skeleton compounds in plants are produced by the manganiferous acid pathway, and the two synthesis pathways of SA and IAA, phenylalanine and tryptophan pathways, are generated from the product chorismic acids in the manganiferous acid pathway. IAA is the signal of anthocyanin accumulation at insect chewing sites and induces systemic acquired resistance [29]. Metabolites can be the mirror of resistant development in plants in vivo. Junji Takabayashi and Kaori Shiojiri demonstrated the impact of feeding-stimulation-induced volatile organic compounds (VOCs) on insects with various feeding habits through field and indoor experiments [30]. However, the correlation between hormones and resistance substances has been mostly explored by applying exogenous hormones indoors. The correlation of the expression of endogenous hormones and resistance in trees under natural condition is still unclear when perceiving a stimulus.

The composition and content of defense enzymes, secondary metabolites, and protease inhibitors in Chinese pine (*Pinus tabuliformis* Carr.) in natural conditions are significantly changed after biotic stress, and the content of chemical substances, such as alkaloids, phenolic compounds, and tannins, increases with the changes of defense proteins [31,32,33,34]. However, these studies only described the changes in individual substances, and there is no holistic understanding of the metabolic pathway of insect resistance in conifers. With the completion of chromosome genome sequencing in Chinese pine, the complex regulatory elements on gene expression also provide a strong reference for the study of insect resistance mechanisms in conifers [35].

Currently, the ecological environment is not optimistic, and an increasing number of scholars have noted the importance of endogenous resistance in stress response [36,37]. The application of omics technology is increasingly becoming extensive in the discovery of molecular mechanism. Though many studies have been conducted and drawn a conclusion on plant resistance, when pests occur in nature, the environment cannot be precise. Furthermore, when facing caterpillar damage, there is no proven method to stifle except chemical reagents. Long life cycles and complex ploidy also make it difficult to study stress resistance in trees [38]. To quantify the overall changes in metabolites during the formation of insect resistance in conifers, we randomly selected Chinese pine and Chinese pine caterpillars (*Dendrolimus tabulaefomis* Tsai et Liu) under natural conditions to determine the expression of the endogenous phytohormones JA, SA, IAA, and ABA after different treatments. We also used clipping as mechanical damage control and conducted metabolome identification and validation for untreated and treated Chinese pine needles. The results revealed metabolite changes in the flavonoid pathway during conifers’ response to insect stimulation and suggest antagonistic interactions between growth- and resistance-related hormones, providing a point of view for related research on other conifers or mandibulate insects.

## 2. Results

### 2.1. Expression of Metabolites of Feeding Stimulation and Leaf Clipping Control

**Figure 1:**
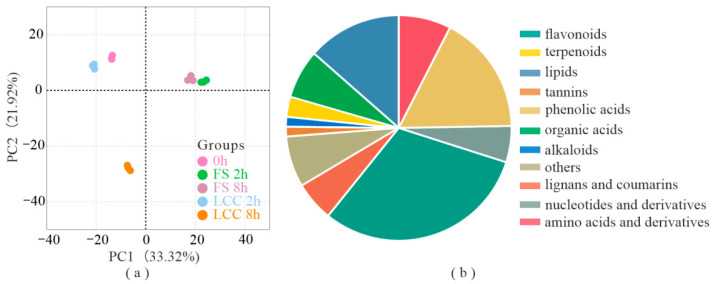
Background of metabolites. (**a**) Principal component analysis (PCA) of metabolome. (**b**) Proportion of different metabolite types in all metabolites.

To further explain metabolite changes in Chinese pine after feeding stimulation, primary and secondary metabolites were identified in the needles at 0, 2, and 8 h after feeding stimulation (marked as FS 2 h and FS 8 h) and leaf clipping control (marked as LCC 2 h and LCC 8 h) using an ultra-performance liquid chromatography–tandem mass spectrometry (UPLC–MS/MS) platform with extensive targeted metabolism techniques. A total of 999 metabolites were identified. Principal component analysis (PCA) was then conducted, and the first principal component (PC1) explained 33.3% of the original data (Figure 1a). All metabolites were clearly separated into five groups, which showed that both treatment modes and time after treatment could lead to the major difference in expression. As shown in Figure 1b, the top 3 were flavonoids (30.8%), phenolic acid (17.2%), and lipids (13.15%). A total of 93 flavonols, 90 flavonoids, 37 dihydroflavones, 19 flavonoid glycosides, 17 flavanols, 15 chalcones, 15 isoflavones, 13 dihydroflavones, 7 anthocyanins, and 2 diflavones were detected.

**Figure 2:**
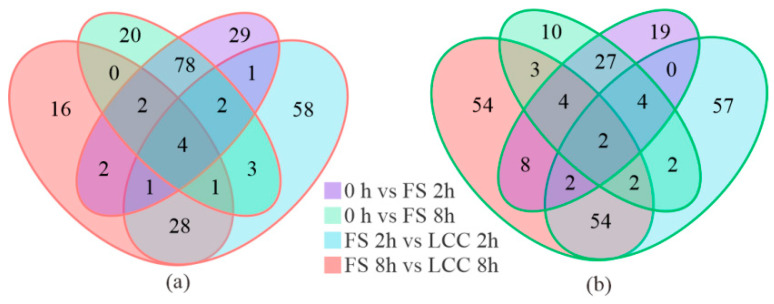
Venn diagram of upregulated differentially accumulated metabolites (DAMs) (**a**) and downregulated DAMs (**b**) in different comparison groups. Different color represents different groups.

Variable importance in projection (VIP), based on the orthogonal partial least squares-discriminant analysis (OPLS-DA) model combined with the *p*-value and fold change (FC) from univariate analysis, was used to further identify differential metabolites. Substances with FC ≥ 2 and ≤ 0.5 and VIP ≥ 1 were selected as differentially accumulated metabolites (DAMs). In the 0 h vs. FS 2 h group and the 0 h vs. FS 8 h group, there were 248 (158 upregulated and 90 downregulated) and 202 (139 upregulated and 63 downregulated) DAMs. A total of 297 DAMs were detected 2 h after treatment, of which 126 metabolites were upregulated and 171 were downregulated. In the comparison at 8 h after treatment, a total of 239 DAMs were detected, of which 62 were upregulated and 177 were downregulated (Table S1). Seventy-eight DAMs were upregulated only in the comparison of 0 h and FS, and 27 DAMs were downregulated (Figure 2a,b). Notably, plant hormone expression was downregulated in both time comparison groups with different treatments.

### 2.2. Functional Enrichment Analysis of DAMs

**Figure 3:**
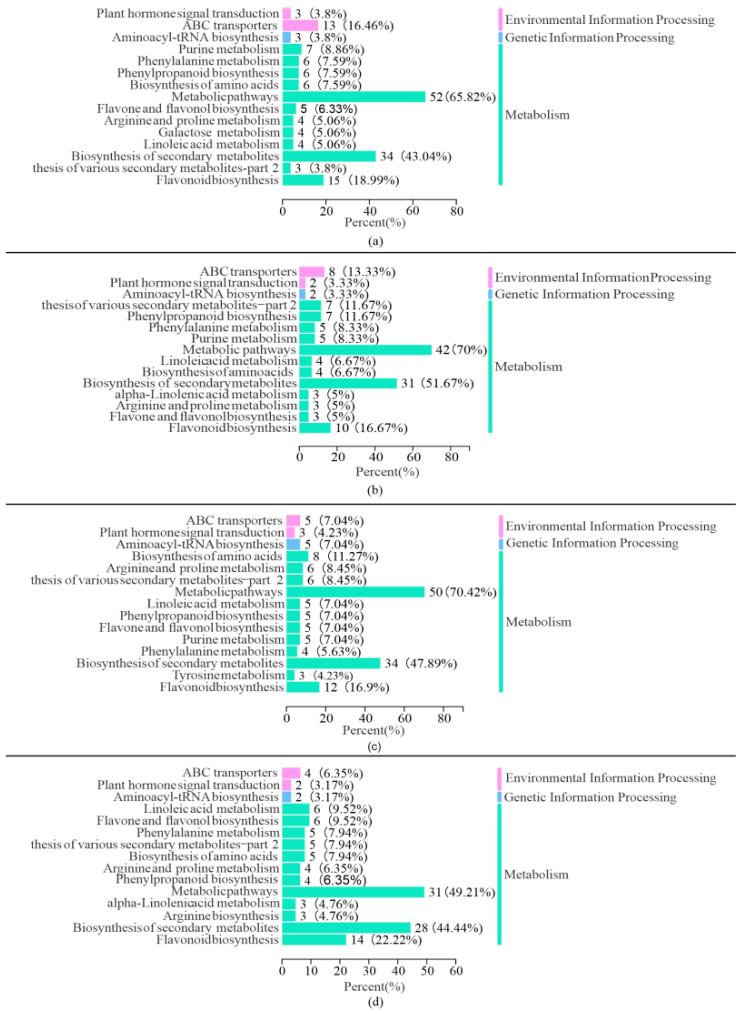
Bar plot of the top 15 clustered KEGG pathways of the DAMs. (**a**) Comparison group of 0 h vs. FS 2 h. (**b**) Comparison group of 0 h vs. FS 8 h. (**c**) Comparison group of FS 2 h vs. LCC 2 h. (**d**) Comparison group of FS 8 h vs. LCC at 8 h. The horizontal coordinate is the percentage of DAMs annotated to the pathway, and the vertical coordinate is the pathway name. Different colors represent different biological processes.

We mapped all DAMs from different comparison groups into the Kyoto Encyclopedia of Genes and Genomes (KEGG) database. After anchoring to the pathways in which metabolites are involved, the annotation results were clustered to identify the pathways in which more DAMs were enriched. As shown in Figure 3, except for the metabolic pathways and biosynthesis of secondary metabolites, the top 3 pathways annotated and enriched for DAMs at FS 2 h vs. LCC 2 h were flavonoid biosynthesis (12), amino acid synthesis (8), and arginine and proline metabolism pathways (6). The top 3 pathways at FS 8 h vs. LCC 8 h were flavonoid biosynthesis (14), linoleic acid biosynthesis (6), and flavonoid and flavonol biosynthesis pathways (6). When considering time as a variable, at 2 h after FS, the top 3 clustered pathways were the flavonoid (15), purine (7), and phenylalanine metabolism pathways (6). At 8 h after caterpillar stress, the top 3 clustered pathways were the flavonoid (10), phenylpropanoid (7), and phenylalanine pathways (5). Plant hormone signal transduction and ATP-binding cassette (ABC) transporters in environmental information processing and aminoacyl-tRNA biosynthesis in genetic information processing were contained in all four comparison groups. Though citric acid and glutathione act as a critical part of defense, there is no significant cluster of metabolites within caterpillar stress.

**Figure 4:**
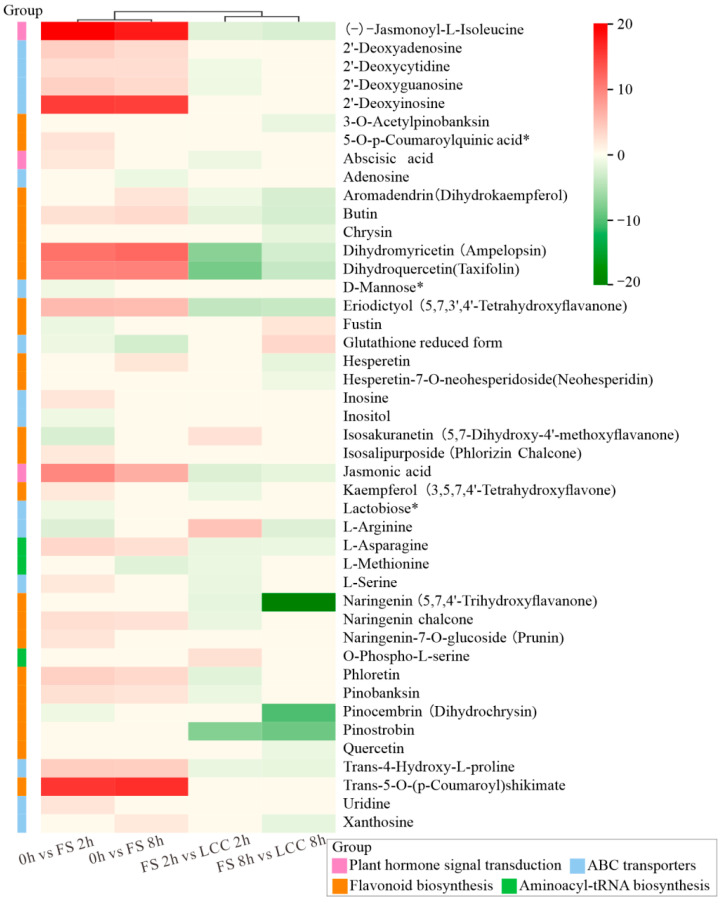
Heat map of Log_2_FC for each differential metabolite reflects the expression between the comparison groups. * in heat map means when using this CAS registry number alone as a research term, it may result in incomplete research results.

We use log_2_FC of DAMs involved in four shared pathways—plant hormone signal transduction, ABC transporters, flavonoid biosynthesis, and aminoacyl-tRNA biosynthesis—to present the expression level. As shown in Figure 4, (-)-jasmonoyl-L-isoleucine, 2′-deoxyinosine, dihydromyricetin (ampelopsin), dihydroquercetin (taxifolin), JA, and trans-5-O-(p-coumaroyl) shikimate exhibited significant upregulation in the 0 h vs. FS 2 h and 0 h vs. FS 8 h comparison groups. LCC led to the downregulation of naringenin (5,7,4′-trihydroxyflavanone), pinocembrin (dihydrochrysin), and pinostrobin.

### 2.3. Content and Correlation Analysis of Plant Hormones

**Figure 5:**
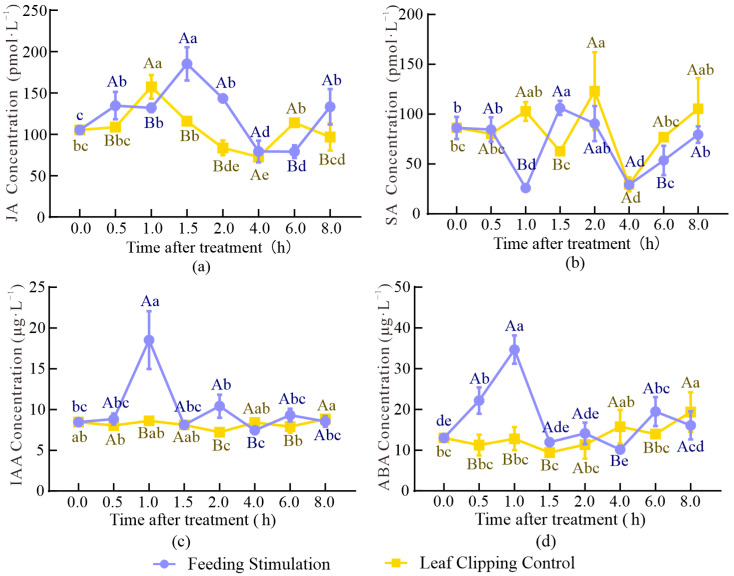
Concentration of JA (**a**), SA (**b**), IAA (**c**), and ABA (**d**) at different times after treatment. The data involved in this article were the mean ± SD (n = 3). Different uppercase letters meant significant differences between different treat modes and the same time after treatment at the *p* < 0.05 level. Different lowercase letters meant significant differences between different time after treatments and the same treat mode at the *p* < 0.05 level.

To characterize the changes in plant hormone content during insect resistance processes in conifers, the plant endogenous hormones JA, SA, IAA, and ABA were selected for the expression assay. The concentrations of the four plant hormones in the samples are shown in Figure 5 and Table S2.

The JA content in FS first increased, then decreased, and increased again over time (Figure 5a). The largest amount of JA accumulation (185.16 pmol·L^−1^) following FS treatment occurred at 1.5 h, which was 59.8% higher than the JA levels at 1.5 h following LCC treatment. The largest amount of JA accumulation (157.43 pmol·L^−1^) following LCC treatment occurred at 1 h, which was 19.2% higher than the JA levels at 1 h. All time points showed a higher JA content in FS than in LCC, except for 1 and 6 h. This showed that JA upregulation was induced in a short time (2 h) after FS compared with LCC.

FS led to a sharp decrease in SA at 1 and 4 h, with contents of 25.86 and 29.21 pmol·L^−1^, respectively, which were 70.0% and 66.1% lower, respectively, than at 0 h. The minimum for LCC occurred only at 4 h after treatment, and the maximum at 2 h after treatment at 115.22 pmol·L^−1^. After 2 h, the SA content for LCC was higher than that for FS at all time points. The content of each treatment showed a significant increase over time, and there were significant differences at 1, 1.5, and 2 h after the two different treatments (Figure 5b).

IAA content reached a peak at 18.53 μg·L^−1^ in 1 h. IAA was significantly downregulated in LCC at 2 h and was significantly different from FS (Figure 5c). At 4 and 8 h, the IAA content following LCC treatment was lower than that following FS treatment, and the content of LCC was lower than that of FS, indicating that IAA was significantly induced by FS in a short time (2 h), but LCC did not cause remarkable changes over time.

FS caused a continuous increase in ABA content to 34.68 μg·L^−1^ for 1 h and then decreased; subsequently, there was an upregulation at 6 and 8 h, with a 49.8% and 23.6% increase over that at 0 h, respectively (Figure 5d). The maximum ABA content with LCC was 19.36 μg·L^−1^, which occurred at 8 h, and a significant difference in expression from 0 h was only observed at 8 h. The minimum content occurred at 1.5 h (9.40 μg·L^−1^). Although there was a significant difference between the two treatments at 4 and 6 h, the content at 4 h for LCC was higher than that in FS, while at 6 h, the two treatments led to opposite results.

Pearson correlation analysis of the four plant hormones at different times showed significant positive correlations between JA and SA (Table S2), as well as between IAA and ABA, with correlation coefficients of 0.36(*p* < 0.05) and 0.77(*p* < 0.01), respectively; SA showed negative correlations with IAA and ABA, with correlation coefficients of 0.37 and 0.34, respectively, at the *p* < 0.05 level.

### 2.4. Content of Total Flavonoid

**Figure 6:**
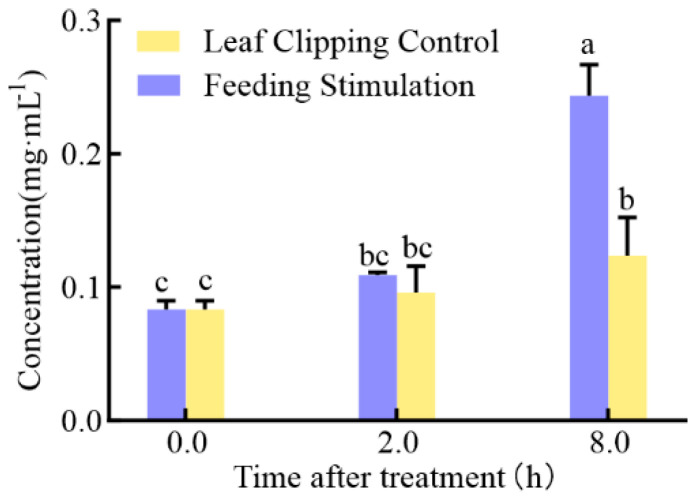
Total flavonoid concentration at different times after treatment. The data involved were the mean ± SD (n = 3). The same letter means there is no significant difference at the *p* < 0.05 level.

To validate the metabolomics results, the total flavonoid content, the top class of substances with the greatest accumulation differences in the metabolome, was determined. As shown in Figure 6, both treatments induced flavonoid accumulation over time. The total flavonoid content was 0.24 and 0.12 mg·mL^−1^ for FS 8 h and LCC 8 h, respectively, which were significantly different from 0.08 mg·mL^−1^ at 0 h. FS treatment induced a highly significant difference in accumulation. There was no significant difference between FS 2 h and LCC 2 h, but a significant difference was observed between FS 8 h and LCC 8 h. There was a significant difference between FS 2 h and FS 8 h, while in LCC, they were not significantly different.

## 3. Discussion

### 3.1. Differential Accumulated Flavonoids in Chinese Pine after FS and LCC

**Figure 7:**
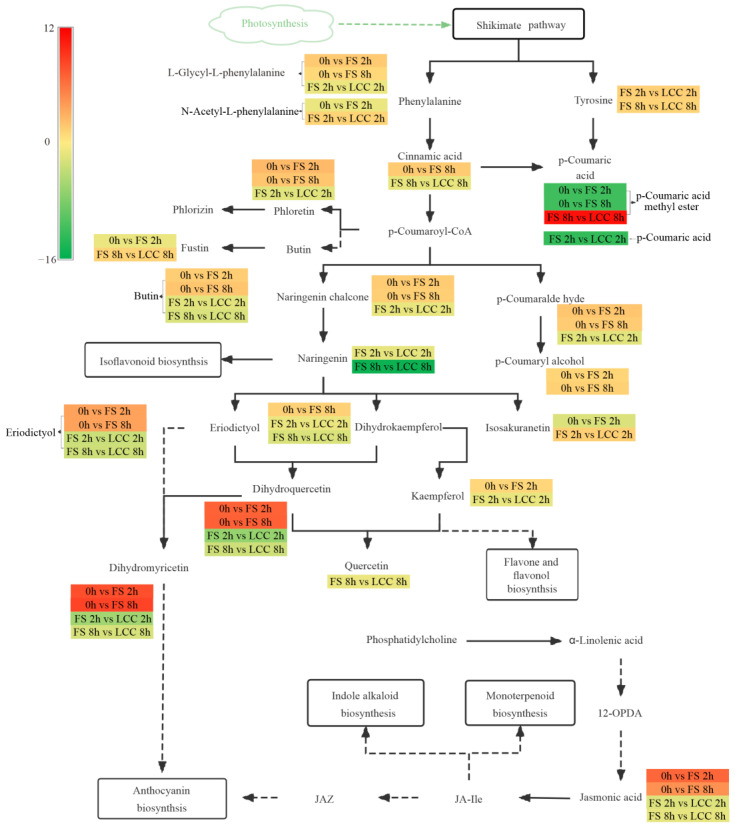
Pathway and expression of flavonoids, phenylpropanoids, and JA with different accumulations. The blocks are the log_2_FC of the response substances, and the color indicates the expression level. Solid lines show the reaction with no intermediate steps, and dashed lines show the reactions requiring an intermediate process.

Stress on plants induces a large number of ROS. Flavonoids in response to stress in plants are mainly involved in the antioxidant scavenging of free radicals [39,40,41]. In this study, a significant amount of DAMs were enriched in the flavonoid pathway in both FS and LCC comparison groups, as shown in Figure 3. According to the KEGG pathway, the C4H cleavage of cinnamic acid to 4-coumaric acid occurs in the phenylalanine pathway [42], which in turn generates p-coumaroyl CoA, followed by naringenin chalcone synthesis catalyzed by chalcone synthase [43], the precursor of most different accumulated flavonoids in this study. This process was upregulated by caterpillars, but there was no difference in expression in FS 8 h vs. LCC 8 h. However, as shown in Figure 7, naringenin, a downstream substance of chalcone, was not induced by FS but was significantly higher in FS than in LCC at 8 h. Therefore, mechanism damage can promote naringin depletion and become more intense over time. The downstream substance of naringenin, eriodictyol, was induced by FS but not by LCC. The longer the feeding stimulation by pine caterpillars, the weaker the reaction of naringenin flow to isosakuranin, causing eriodictyol and others to form flavanonol, dihydrokaempferol, dihydroquercetin, and dihydromyricetin catalyzed by flavanone 3-hydroxylase (F3H) [43]. There were more significant differences in dihydromyricetin and dihydroquercetin at 2 h than at 8 h for different treatment modes, but the opposite trend was observed in aromadendrin (dihydrokaempferol) accumulation. Furthermore, no differences were observed in its downstream substance, kaempferol, between the FS and LCC comparison groups at 8 h. This indicates that anthocyanin may play a key role in resistance. In alfalfa, graphene induces the upregulation of isoquercitrin and proline, which are involved in the plant scavenging of ROS to protect them from oxidative damage [44]. At 8 h after treatment, pine caterpillars induced the dihydrokaempferol–dihydroquercetin–quercetin reaction, and at the same time, kaempferol was involved in flavonoid and flavonol biosynthesis (Table S1). These results are consistent with metabolite changes in sugarcane after stickleback feeding and in tea trees after looper feeding [10,45].

FS also induced a significant upregulation of dihydromyricetin and dihydroquercetin, which are involved in anthocyanin biosynthesis (Figure 3). The expression of JA and anthocyanin-related genes in maize (*Zea mays* L.) was highly upregulated by whitefly [*Bemisia tabaci* (Genn.)] attack [46]. Anthocyanin in corn can affect the growth and development of tobacco hornworm (*Manduca sexta* L.) caterpillars [47]. In *Arabidopsis thaliana*, JA releases WD-repeat/bHLH/MYB complexes to accumulate anthocyanin biosynthesis [48]. Therefore, anthocyanins, as flavonoids, react to stress in plants with JA. In this study, the JA concentration reached its peak 1 h after FS, confirming this statement.

In this study, the total flavonoid concentration was determined to validate the metabolomic results. A one-way ANOVA on total flavonoid content revealed changes in flavonoid content due to different treatments and the time after treatment, with a significant difference between FS and LCC treatments at 0 and 8 h, indicating significant differences due to feeding stimulation. These results can not only validate metabolomic results but also indicate that other behaviors or substances of insects, in addition to mechanical damage from feeding, could trigger a defense response.

### 3.2. Differential Accumulated Phenylpropanoid-Derived Compounds in Chinese Pine after FS and LCC

Flavonoids are derived from phenylalanine, and their expression can be induced by insect herbivory. In plants, phenylalanine deaminates to cinnamic acid using phenylalanine ammonia-lyase (PAL), which is the first step in generating other phenylpropanoid-derived compounds [49], considered a bridge between primary metabolism and the synthesis of lignin, flavonoids, or other resistance substances during defense [50]. Phenylpropanoid-derived compounds widely participate in the response to biotic stress. Genes in the phenylpropanoid pathway are upregulated in *Ulmus minor* by an egg treatment of the elm leaf beetle (*Xanthogaleruca luteola*) [51]. PAL can increase SA content to regulate the plant–cereal cyst nematode (CCN) interaction [52]. As shown in Figure 2, more DAMs were enriched in the phenylalanine metabolism and phenylpropanoid biosynthesis pathways in the 0 h vs. FS 2 h and 0 h vs. FS 8 h comparison groups. Coniferyl aldehyde was upregulated after FS. *p*-Coumaric acid, caffeic acid, p-coumaraldehyde, and conifer alcohol were depressed at LCC 2 h, while cinnamic acid and cinnamaldehyde were depressed at LCC 8 h. Precursors of guaiacyl lignin, coniferyl-aldehyde, and conifer-alcohol also accumulated differently after FS (Table S1). As shown in Figure 3, the phenylpropanoid pathway was enriched in four comparison groups, which means that its mechanical damage leads to the accumulation of phenylpropanoid. *p*-Coumaric CoA is related to the phenylalanine metabolism and flavonoid metabolism pathways, as shown in Figure 7, and accumulated under FS, although the change was not significant. As mentioned previously, anthocyanin is regulated by JA, and changes in phenylalanine may occur earlier than 2 h after stress.

Coumarins are also resistant substances derived from the phenylpropanoid pathway [53,54], and scopoletin could eliminate superoxide anions in vivo [55], especially in fungus infection [56]. Surprisingly, scopoletin showed downregulation at FS 8 h vs LCC at 8 h. Although scopoletin can reduce the larval weight of *Spodoptera frugiperda* (J. E. Smith) [57] and induce calcium overload in *T*. *cinnabarinus* [58], it was downregulated by feeding stimulation of pine caterpillar, and whether this process relies on jasmonate signaling should be further studied.

### 3.3. Changes in Plant Hormones in Response to FS and LCC

Plants immediately synthesize considerable JA once they perceive stimulation, and jasmonoyl isoleucine can bind to COI1 receptors to mediate the ubiquitination and degradation of JAZs, thereby activating JA signaling pathways [59]. In this study, JA and jasmonoyl isoleucine accumulated differentially at 2 and 8 h in both treatments and were higher for FS than for LCC, which is consistent with the changing trend of plant hormones. JA content increased rapidly in the short term during feeding stimulation and then decreased to a level with no significant difference in expression from the pretreatment (Figure 4), avoiding excessive plant defenses that hindered its growth and development due to excessive JA accumulation. The regulation of SA in plant-chewing insect interaction is always accompanied by other plant hormones, such as JA. In this study, there was no significant difference in SA content between FS and LCC, and the expression of related substances was not detected in metabolome; however, the long duration of feeding stimulation led to changes in SA content. This study showed that in the tryptophan pathway, indole produces not only IAA but also 2-carboxamidobenzoate, but this reaction was only seen in the comparison group with short-term treatments (2 h) (Table S1). Although there was no difference between SA and IAA accumulation in either comparison group of the metabolome, the difference between these two substances showed a negative correlation at 1 h after different treatments. The IAA concentration was upregulated before JA, which is consistent with a previous study [29]. Thus, enzymes in the heterobranched acid pathway may play an important role in balancing defense and growth, which need further research. As shown in Table S1, γ-aminobutyric acid (GABA) was upregulated 2 h after feeding stimulation. ABA signaling pathway activation can be observed by applying GABA to water-stressed apple shoots [60]. Meanwhile, GABA can activate the gene expression of ACC, the precursor of ethylene synthesis, and the ethylene-induced root growth requires IAA biosynthesis, transport, signaling, and response. The same positive correlation between IAA and ABA can be induced by feeding stimulation of pine caterpillars. In previous studies, chewing insects induce the coregulated resistance formation of JA and ABA. The downregulation of ABA signaling induced by brown planthoppers can increase SA-dependent defense in response to salt stress in susceptible rice varieties [61]. Similarly, the increase in ABA and JA content in rice after silencing E3 ligase also demonstrates brown planthopper-induced plant hormone antagonism [62]. Correlation analysis indicated a correlation between SA and ABA, but the lower significance of these plant hormones induced by the feeding stimulation of pine caterpillars on Chinese pine instead of high significance may occur because of the foraging pattern; that is, the brown planthopper feeds on the phloem, where ABA transport occurs over a long distance.

## 4. Materials and Methods

### 4.1. General Situation of the Research Area

A pure Chinese pine forest with the same elevation, aspect, and slope, as well as a similar tree vigor and management level, was selected in Huangtuliangzi Forestry, Pingquan City, Chengde City, Hebei Province, China (41°18′ N, 119°13′ E). The plants were maintained under natural conditions. The forest is located in the northeastern part of Hebei Province, adjacent to Inner Mongolia and Liaoning Provinces, and has a midtemperate continental dry monsoon mountain climate with an annual average temperature of 6.6 °C, a frost-free period of 120–130 days and a rainfall of about 540 mm.

### 4.2. Treatment Modes

Chinese pine caterpillars underwent starvation for 10 h before conducting the experiment. A total of 75 healthy trees about 10 years old without any herbivorous insect stress were randomly selected and grouped into 15 groups at different times per treatment, with a replication of 5 trees in each group. Branches with consistent branch length and height above the ground were selected in four directions: east, west, north, and south of each tree. The treatment groups were given feeding stimulation (FS) by 10 caterpillars and leaf clipping control (LCC) by repeatedly cutting off needle tips for mechanical damage simulation foraging by 10 caterpillars, while the control (CK, recorded as 0 h) was not given any treatment. In the treatment group, 10 pine needles were collected from each branch as one parcel at 0.5, 1, 1.5, 2, 4, 6, and 8 h after treatment, and the control group contained 10 intact pine needles as one parcel. Needles were preserved in tinfoil, frozen in liquid nitrogen, and transferred to −80 °C for storage. A total of 20 parcels of pine needles were acquired from 5 trees at different times under each treatment.

### 4.3. Metabolomic Profiling Analysis

Pine needles were freeze-dried using a vacuum freeze dryer (Scientz-100F). The freeze-dried sample was crushed using a mixer mill (MM 400, Retsch) with a zirconia bead for 1.5 min at 30 hz. Lyophilized powder (100 mg) was dissolved in 1.2 mL of 70% methanol solution and vortexed six times for 30 s every 30 min. The samples were then refrigerated at 4 °C overnight. Following centrifugation at 12,000 rpm for 10 min, the extracts were filtrated (SCAA-104, 0.22 μm pore size; ANPEL, Shanghai, China, http://www.anpel.com.cn/) before UPLC–MS/MS analysis.

The analytical conditions were as follows: UPLC: column, Agilent SB-C18 (1.8 µm, 2.1 mm * 100 mm); the mobile phase consisted of solvent A, pure water with 0.1% formic acid, and solvent B, acetonitrile with 0.1% formic acid. Sample measurements were performed with a gradient program that employed the starting conditions of 95% A, 5% B. Within 9 min, in a linear gradient to 5% A, 95% B was programmed, and in a composition of 5% A, 95% B was kept for 1 min. Subsequently, in a composition of 95% A, 5.0% B was adjusted within 1.1 min and kept for 2.9 min. The flow velocity was set as 0.35 mL per minute, the column oven was set to 40 °C, and the injection volume was 4 μL. The effluent was alternatively connected to an ESI-triple quadrupole-linear ion trap (QTRAP)-MS.

LIT and triple quadrupole (QQQ) scans were acquired on a triple quadrupole-linear ion trap mass spectrometer (Q TRAP), AB4500 Q TRAP UPLC/MS/MS System, equipped with an ESI Turbo Ion-Spray interface, operating in positive and negative ion mode and controlled by the Analyst 1.6.3 software (AB Sciex). The ESI source operation parameters were as follows: ion source, turbo spray; source temperature, 550 °C; ion spray voltage, (IS) 5500 V (positive ion mode)/-4500 V (negative ion mode). Ion source gas I (GSI), gas II (GSII), and curtain gas (CUR) were set at 50, 60, and 25.0 psi, respectively; the collision-activated dissociation (CAD) was high. Instrument tuning and mass calibration were performed with 10 and 100 μmol/L polypropylene glycol solutions in QQQ and LIT modes, respectively. QQQ scans were acquired as MRM experiments with collision gas (nitrogen) set to medium. DP and CE for individual MRM transitions were performed with further DP and CE optimization. A specific set of MRM transitions was monitored for each period according to the metabolites eluted within this period.

In all identified 999 compounds (Table S3), significantly regulated metabolites between groups were determined by VIP we1 and absolute log2FC (fold change) ≥ 1. VIP values were extracted from the OPLS-DA result, which also contained score plots and permutation plots, generated using the R package MetaboAnalystR. The data were log transform (log2) and mean centering before OPLS-DA. In order to avoid overfitting, a permutation test (200 permutations) was performed.

### 4.4. Determination of Plant Hormone Content

Pine needles (0.6 g) were well milled in liquid nitrogen. Then, 5.4 mL of PBS buffer (pH = 7.3) was added and centrifuged for 30 min at 5000 r/min at 4 °C. The contents of JA, SA, IAA, and ABA were determined using Plant Hormone ELISA Kits (Shanghai Enzyme-linked Biotechnology Co., Ltd., Shanghai, China). The kit employed a double antibody sandwich method to determine the levels of plant hormones in the sample. The optical density values were measured at 450 nm with a microplate reader, and the standard curves were fitted in ELISA Calc by a four-parameter logistic model. All *r*^2^ values were higher than 0.9, indicating a good correlation with the model.

### 4.5. Determination of Flavonoid Content

Pine needles (0.6 g) were ground in liquid nitrogen, and 6 mL of extraction solution was added. Samples were then submitted to 300 W ultrasonication extract (60 °C) in 8 s intervals every 5 s for 30 min and then centrifuged at 12,000 rpm for 10 min at 25 °C. The flavonoid content was determined using a plant flavonoid content assay kit (Beijing Boxbio Science & Technology Co., Ltd., Beijing, China). The optical density values were measured at 510 nm with a microplate reader, and the flavonoid content in plants was calculated. The standard curve was y = 0.6457 x − 0.0059, and *r*^2^ was higher than 0.99.

### 4.6. Data Processing

The statistics involved in this article were the mean ± SD from three biological replications. Plant hormone concentrations were tested using Duncan’s new complex polar difference method and analysis with two-way ANOVA. Total flavonoid assay results were tested using the LSD method and analysis with one-way ANOVA. All data were calculated by SPSS 21.0. The significance was determined at *p* < 0.05 except as specifically mentioned.

## 5. Conclusions

This study reports the change of metabolome and the content of JA, SA, IAA, and ABA in Chinese pine after feeding stimulation by Chinese pine caterpillar. It was found that Chinese pine can trigger the flavonoid pathway, compared with JA, IAA, and ABA following caterpillar feeding and mechanical wounding. Our metabolome data offered insights into the response to caterpillar herbivory and wounding of pine needles. Further research is required to understand the balance between pine defense responses to herbivory and the impact on tree growth.

## Data Availability

Not applicable.

## Supplementary Materials

The following supporting information can be downloaded at: https://www.mdpi.com/article/10.3390/cimb45020072/s1, Table S1: DAMs of the four group: 0 h vs. FS 2 h, 0 h vs. FS 8 h, FS 2 h vs. LCC 2 h, and FS 8 h vs. LCC 8 h. Table S2: Concentration and Pearson correlation analysis of four plant hormones. Table S3: Identify information of all 999 compounds.
